# Time required to achieve the minimal clinically important difference after open proximal hamstring repair

**DOI:** 10.1093/jhps/hnae045

**Published:** 2025-01-29

**Authors:** Alexander E White, Nathan H Varady, Thun Itthipanichpong, Samarth V Menta, Anil S Ranawat

**Affiliations:** Sports Medicine Institute, Hospital for Special Surgery, 535 E 70th Street, New York, NY 10021, United States; Sports Medicine Institute, Hospital for Special Surgery, 535 E 70th Street, New York, NY 10021, United States; Sports Medicine Institute, Hospital for Special Surgery, 535 E 70th Street, New York, NY 10021, United States; Sports Medicine Institute, Hospital for Special Surgery, 535 E 70th Street, New York, NY 10021, United States; Sports Medicine Institute, Hospital for Special Surgery, 535 E 70th Street, New York, NY 10021, United States

## Abstract

Understanding the minimal clinically important difference (MCID) for a given procedure and its associated patient-reported outcome measures (PROMs) are critical for evaluating success in orthopedic surgery. The MCIDs for the International Hip Outcome Tool (iHOT-33) and Modified Harris Hip Score (mHHS) have been defined for open proximal hamstring repair (OPHR); however, the speed and reliability at which patients achieve these are unknown. A retrospective review of prospectively collected data from our institution’s hip preservation registry was performed, examining pre-operative and 6-, 12-, and 24 months post-operative mHHS and iHOT-33 scores. The percentage of patients achieving MCID at each time point was determined, and factors associated with achieving MCID were assessed. A total of 37 patients were included in this analysis (*n* = 36 for iHOT-33 and *n* = 32 for mHHS). At 6 months, 83% and 78% of patients achieved MCID for iHOT-33 and mHHS, respectively. Patients with chronic symptoms (pain >6 months) were significantly less likely to achieve at least one of the MCIDs at 6 months (60% vs. 12.5%, *P* = .04), while patients with more severe preoperative pain were significantly more likely to achieve at least one of the MCIDs at 6 months (*P* = .004). Most patients who achieve the MCID for iHOT-33 and mHHS following OPHR do so by 6 months postoperatively. Chronic symptoms were associated with failure to achieve either one of the MCIDs at 6 months post-operatively. Patients with more severe preoperative pain were more likely to successfully achieve one of the MCIDs at 6 months.

## Introduction

Hamstring strains account for 25–30% of all muscle strains, making it the most commonly injured muscle in the body. Up to 12% of proximal hamstring strains involve a tear or avulsion at the proximal hamstring origin [1–3]. Non-operative treatment for proximal hamstring avulsions or tears can result in intractable pain and significant morbidity. As a result, acute surgical repair of the proximal hamstring is recommended when possible. Numerous studies have demonstrated that proximal hamstring repair can successfully return patients to sport [4–6] and yield higher function and strength compared to non-operative management as reflected in patient-reported outcome measures (PROMs) [7].

As the use of PROMs becomes more ubiquitous in orthopedics, the interpretation of these measures also continues to evolve. The minimal clinically important difference (MCID) has become an essential benchmark in recent orthopedic PROMs literature [8, 9]. In fact, a recent study defined the MCID for open proximal hamstring repairs when considering the International Hip Outcome Tool (iHOT-33) and modified Harris Hip Score (mHHS) [10]. However, the probability of achieving the MCID at different timepoints following open proximal hamstring repair has not yet been determined. The time to achieving various clinically significant outcomes has been studied extensively in the orthopedic literature as it provides valuable prognostic information for surgeons and patients alike [11–13]. Studies have previously identified 6 months post-operatively to be an important inflection point for patients achieving the MCID for several common PROMs following rotator cuff repair and hip arthroscopy among other surgeries [12, 13].

The purpose of this study is to determine the probability of achieving the MCID for iHOT-33 and mHHS after open proximal hamstring repair at 6-, 12-, and 24-month follow-up. A secondary aim of this study was to identify patient characteristics that might predict a likelihood of achieving MCID post-operatively. It was hypothesized that greater than 80% of patients would achieve MCID at 6-month follow-up after open proximal hamstring repair and that the percentage to achieve MCID would not meaningfully improve beyond 6 months.

## Materials and methods

This retrospective review of prospectively collected data aimed to determine the probability of achieving the MCID for the iHOT-33 and mHHS at 6-, 12-, and 24-month time points following open proximal hamstring repair. The data were obtained from our institution’s hip preservation registry. Surgeries were performed by three separate fellowship-trained orthopedic surgeons. Patients were included if they were above 18 years of age, underwent primary open proximal hamstring repair for a diagnosis of hamstring tear at our institution, had a minimum follow-up period of 6 months, and completed PROMs pre-operatively and post-operatively. Patients were excluded if they had a history of prior ipsilateral hip surgery, a pre-existing congenital hip condition, high-grade osteoarthritis (Tonnis grade ≥ 2), or underwent concomitant cartilaginous or realignment procedures.

The proximal hamstring repair procedure is performed in the prone position, and the entire lower extremity is prepped and draped in a usual sterile fashion. An incision is carried down through the subgluteal fascia with special care to protect the cluneal and sciatic nerves. The hamstring fascial sheath is incised and any present hematoma is evacuated. Next, the ischial tuberosity is identified and curetted down to bleeding bone. Typically, suture anchors are then placed into the ischial tuberosity, and the loaded sutures are passed through the proximal hamstring tendon in a combined whipstitch and mattress-type configuration and tied down.

Demographic information (including age, sex, body mass index, and laterality), baseline patient factors (including baseline pain severity and duration of symptoms), procedures performed, complications, and PROMs were collected from the hip registry. The primary outcome of interest was the percentage of patients achieving the MCID at each time point after surgery. The MCID values for open proximal hamstring repairs, as established in previous studies, were defined as an improvement of 12.6 and 11.8 in theiHOT-33 and mHHS, respectively, from the preoperative baseline [10]. Patients with baseline iHOT-33 or mHHS values greater than 100 minus the respective MCID threshold (i.e. baseline iHOT-33 > 87.4 and/or mHHS > 88.2) were excluded from the analysis of that PROM, as these patients could not have possibly achieved MCID.

### Statistical analysis

Descriptive statistics were used to illustrate the probability of patients achieving the MCID at each time point. Data were collected on patients’ preoperative pain duration and severity, as well as demographic factors such as sex, age, and BMI. Univariate analyses, including chi-square tests or Fisher’s exact tests for categorical variables and independent t-tests or Wilcoxon Mann-Whitney tests for continuous variables, were conducted to compare baseline factors to the primary outcome of MCID achievement as appropriate. Correlations between PROM improvements at 6 months and continuous factors found to be significant in the primary analysis were also calculated. All eligible patients were included in the data analysis and, therefore, no *a prior* power analysis was performed. Statistical analysis was performed in SAS v9.4 (SAS Institute, Cary, NC), and *P* < .05 was considered significant.

## Results

A total of 37 patients met inclusion criteria for complete analysis. Thirty-six (97%) patients had complete data with iHOT-33 scores, 35 (95%) patients had complete data for mHHS scores, and 34 (92%) patients had both iHOT-33 and mHHS scores. After excluding the three patients who could not have achieved MCID for mHHS (no patients had baseline iHOT-33 scores that would preclude MCID), there were 32 patients included in the analysis of mHHS and 31 patients with data for both mHHS and iHOT-33. Patients were 57% male, with a mean age of 53.3 years (49.4–57.1) and a mean BMI of 27.3 (25.6–29.1) (Table 1). There were 16 left hips and 21 right hips. Mean (95% confidence interval) improvements in iHOT-33 and mHHS scores at 6 months were 36.4 (29.2–43.7) and 26.6 (18.5–34.6), respectively. No patients experienced a complication.

**Table 1.** Baseline demographic factors.

Baseline factor	Result
**Age (years)**	53.3 (49.4–57.1)
**Sex**	
Female	16 (43.2%)
Male	21 (56.8%)
**BMI (kg/m^2^)**	27.3 (25.6–29.1)
**Laterality**	
Left	16 (43.2%)
Right	21 (56.8%)
**Pain duration**	
<3 months	25 (67.6%)
3–6 months	5 (13.5%)
6–12 months	4 (10.8%)
>12 months	3 (8.1%)
**Baseline pain severity**	41.5 (32.5–50.5)

Data presented as mean (95% confidence interval) or *n* (%).

BMI = body mass index.

At 6 months, 87% (32 of 37) of patients achieved the MCID for at least one of iHOT-33 or mHHS, including 83% of patients (30 of 36) for iHOT-33 and 78% of patients (25 of 32) for mHSS (Table 2). Seventy-four percent (23 of 31) of patients achieved MCIDs for both iHOT-33 and mHHS. At 12 months, 86% (18 of 21) of patients achieved the MCID for at least one of iHOT-33 or mHHS, including 80% of patients (16 of 20) for iHOT-33 and 88% of patients (15 of 17) for mHHS. Eighty-one percent (13 of 16) of patients achieved MCIDs for both iHOT-33 and mHHS. At 24 months, 78% of patients (7 of 9) achieved the MCID for at least one of iHOT-33 or mHHS, including 78% of patients (7 of 9) for the iHOT-33 and 71% of patients (5 of 7) for the mHHS.

**Table 2.** Patients achieving minimal clinically important difference (MCID) in iHOT-33 and mHHS at each time point.

Timepoint	*n* (%)
**iHOT-33**	
6 months	32 (86.5%)
12 months	16 (80.0%)
24 months	7 (77.8%)
**mHHS**	
6 months	25 (78.1%)
12 months	15 (88.2%)
24 months	5 (71.4%)
**Either**	
6 months	32 (86.5%)
12 months	18 (85.7%)
24 months	7 (77.8%)
**Both**	
6 months	23 (74.2%)
12 months	13 (81.3%)
24 months	5 (71.4%)

**Figure 1.**
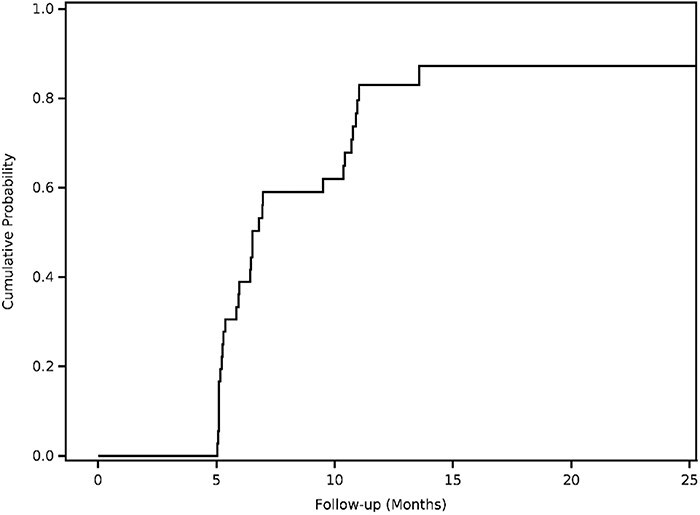
Cumulative probability for achieving minimal clinically important difference (MCID) on the International Hip Outcome Tool (iHOT) score as a function of time.

**Figure 2.**
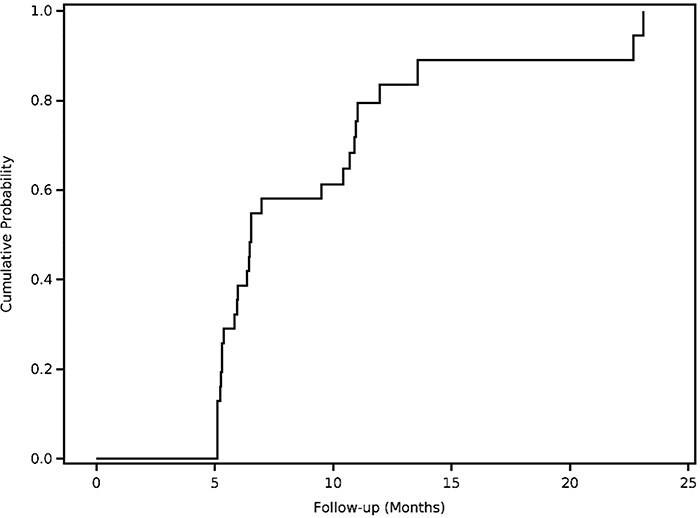
Cumulative probability for achieving minimal clinically important difference (MCID) on the modified Harris Hip Score (mHHS) as a function of time.

Of the six patients who failed to achieve MCID in iHOT-33 at 6 months, four (67%) had 12-month follow-up data, none of whom achieved MCID in iHOT-33 at 12 months (0%) or 24 months (0%). Of the seven patients who failed to achieve the MCID for mHHS, four (57%) had 12-month follow-up data, of whom 3 (75%) achieved the MCID for mHHS at 12 months. No patients further progressed to achieve the MCID in mHHS at 24 months if they had not already done so by 12 months (Figs 1 and 2).

Patients who failed to achieve at least one MCID at 6 months were significantly more likely to have had a preoperative pain duration of >6 months (60% vs. 12.5%, *P* = .04), while patients who achieved at least one MCID at 6 months had significantly greater median (interquartile range) pain severity at baseline (47.0 (23.5–58.5) vs. 6.0 (0.0–15.0), *P* = 0.011) (Table 3). Relatedly, increasing severity of baseline pain was correlated with significantly greater improvement in iHOT-33 (*R *= 0.51, *P* = .004) and mHHS (*R *= 0.55, *P* = .003) at 6 months, while increasing pain duration was correlated with significantly worse improvements in mHHS at 6 months (*R *= −0.48, *P* = .006).

**Table 3.** Baseline factors stratified by whether patient achieved minimal clinically important difference (MCID) by 6 months.

	Achieved MCID for at least one outcome measure at 6 months		
Baseline factor	No (*N* = 5)	Yes (*N* = 32)	*P* value
**Age (years)**	51.8 (20.9–82.6)	53.5 (49.7–57.2)	0.78
**Sex**			0.06
Female	0 (0.0%)	16 (50.0%)	
Male	5 (100.0%)	16 (50.0%)	
**BMI (kg/m^2^)**	28.0 (24.8–31.2)	27.3 (25.3–29.2)	0.79
**Laterality**			
Left	2 (40.0%)	14 (43.8%)	1
Right	3 (60.0%)	18 (56.3%)	
**Baseline pain >6 months**	3 (60.0%)	4 (12.5%)	0.037
**Baseline pain severity^a^**	6.0 (0.0–15.0)	47.0 (23.5–58.5)	0.011

Data presented as mean (95% confidence interval) or *n* (%) unless specified.

BMI = body mass index.

a Presented as median (interquartile range) due to normality criteria.

## Discussion

Proximal hamstring tears are devastating injuries that can cause significant pain and morbidity if left untreated [4, 7, 14]. While proximal hamstring repair is an accepted treatment for these injuries when possible, data on how reliably and how quickly patients achieve statistically meaningful improvements in clinical outcomes were lacking. The most important findings of the present study are that the vast majority (78%–87%) of patients undergoing proximal hamstring repair achieve clinically important outcomes after surgery, as reflected by achieving MCIDs in iHOT-33 and mHHS. Moreover, we found that these improvements occur relatively quickly, with most patients achieving these results by 6 months postoperatively. Interestingly, we also observed that patients who achieved greater improvement in iHOT-33 and mHHS had significantly more preoperative pain, while those who failed to achieve MCID were significantly more likely to have had baseline pain for >6 months. These results add to the large body of evidence supporting surgical repair of proximal hamstring injuries and provide key insights to surgeons and patients regarding preoperative expectations and postoperative management.

The high proportion of patients achieving MCIDs after proximal hamstring repairs adds an additional layer of support for the potential benefits of this procedure when clinically indicated. Among 40 patients undergoing acute proximal hamstring repair, Cohen et al. [15] previously found average Lower Extremity Functional Scores (LEFS) and Marx scores of 95% and 10.0, respectively. In a series of 64 patients undergoing proximal hamstring repair at a minimum of 2-year follow-up, Arner et al. [16] reported average postoperative LEFS of 96% and Marx score of 12.4. This study builds on these results by demonstrating that not only are average PROM scores high postoperatively after hamstring repair but that on a patient-level, a high proportion of patients experience a clinically meaningful level of improvement from their baseline. Moreover, the rate of MCID achievement seen for proximal hamstring repairs in this study (78%–87%) can be benchmarked by comparison to MCID rates seen for other procedures, such as hip arthroscopy (79%) [8] and endoscopic gluteus minimus repair (78%) [9]. Taken together, these results underscore that proximal hamstring repair results in clinically meaningful outcomes for patients.

Importantly, this study demonstrates that the vast majority of patients experience the MCID do so within 6 months. These results are relevant for both patients and surgeons in term of preoperative counseling and expectation setting. By having both a realistic time-course for and probability of achieving a clinically meaningful improvement outcome, patients are well-known to be significantly more satisfied with their operation [17]. The finding that most patients have reached an MCID by 6 months may also be useful in the development and/or refinement of postoperative physical therapy protocols, which have been shown to be highly inconsistent between institutions for proximal hamstring repairs [18]. Furthermore, while we found that a high proportion of patients achieved MCID by 6 months, we also found that if patients do not achieve an MCID by 6 months, they are unlikely to do so down the line. Specifically, no patients who failed to achieve MCID in iHOT-33 by 6 months did so by 12 months; only 3 patients who failed to achieve MCID in mHHS at 6 months did so by 12 months, and no patients progressed to MCID in either iHOT-33 or mHSS beyond 12 months. These data may be useful for surgeons when considering what to do for patients who are not meaningfully improved by 6 months. For instance, as these patients may be unlikely to continue to progress, it may be appropriate to consider additional intervention, whether surgical or otherwise, rather than continued observation at this time.

Interestingly, we found that a higher degree of preoperative pain was associated with significantly greater improvement in outcomes, while a longer duration of symptoms preoperatively was associated with significantly worse outcomes. The fact that worse preoperative pain was associated with greater improvement in outcomes is consistent with a large body of work in orthopedics (primarily from the arthroplasty literature), demonstrating that worse preoperative pain and function is associated with greater improvements in PROMs [19, 20]. This phenomenon is likely due to the fact that the patients with the worst symptoms preoperatively have the most to gain from surgical treatment, as well as more realistic expectations for their outcomes postoperatively. The association between a longer duration of preoperative pain and worse clinical improvement is also consistent with prior literature, which has demonstrated significantly worse outcomes for chronic injuries [21, 22]. For example, in a review of 41 patients by Sarimo et al. [23], the 29 patients with good or excellent results had a mean time to surgery of 2.4 months compared to a mean time to surgery of 11.7 months for the 12 patients experiencing moderate or poor results (*P* < .001). Similarly, in a review of 132 proximal hamstring repairs, Barnett et al. [24] reported that patients with acute injuries were significantly more likely than those with chronic injuries to rate their outcome as good or excellent (*P* = .04). The findings of the present study are consistent with these findings, as patients with injuries >6 months were significantly less likely to achieve MCID postoperatively than those undergoing surgery within 6 months of injury. While some studies have also shown good outcomes with chronic injuries [1, 25, 26], the trend towards improved outcomes with earlier treatment may be related to fibrosis of the avulsed stump and the development of surrounding scar tissues [21].

Although this is the first study to assess how reliably and how quickly patients achieve MCIDs after proximal hamstring repair, it is not without limitations. First, as hamstring repair surgery is relatively rare compared to other sports medicine procedures, our sample size was inherently small, limiting our power to assess for additional factors that may be associated with PROM improvement after hamstring repair surgery, limiting our multivariable analysis. Next, these data were derived from patients at a single institution and generalizability may vary, particularly if performed by lower volume providers. The lower collection rates for PROM data at 12 and 24 months is also a limitation; however, given the vast majority patients who achieved MCIDs by 6 months, this would be unlikely to significantly impact results. Moreover, while many studies with similar sample sizes have reported postoperative outcome data, few have had baseline preoperative data from which PROM improvements and MCIDs can be calculated as was present in this study. Of note, three patients with mHHS data had to be excluded due to high baseline values that would have made it impossible to achieve MCID. In the hip arthroscopy literature, the mHHS has been criticized for ceiling effects [27, 28]; our results suggest that this may also be a limitation of mHHS for proximal hamstring repair. Additionally, there is little consensus on the best outcome measure for evaluating patients after proximal hamstring repair; however, the present study does not include evaluation of the Perth Hamstring Assessment Tool (PHAT), which is the only validated outcome measure for proximal hamstring repair. Finally, as a retrospective study, causality between the statistical relationships seen in this study cannot be concluded; these are only associations [29].

## Conclusion

The vast majority of patients achieve the MCID for iHOT-33 and mHHS following open proximal hamstring repair, with most patients doing so by 6 months postoperatively. In addition, a preoperative pain duration of greater than 6 months was associated with failure to achieve MCID at 6 months, and increased preoperative pain severity is associated with significantly greater improvements in mHHS and iHOT-33 at 6 months. Moving forward, these data may be useful for both patients and surgeons in terms of setting preoperative expectations and guiding postoperative management.

## Data Availability

The data underlying this article will be shared on reasonable request to the corresponding author.
